# Contested solidarity and vulnerability in social media-based public responses to COVID-19 policies of mobility restrictions in Singapore: a qualitative analysis of temporal evolution

**DOI:** 10.1186/s12889-021-12316-0

**Published:** 2021-12-08

**Authors:** Val Alvern Cueco Ligo, Cheng Mun Chang, Huso Yi

**Affiliations:** Saw Swee Hock School of Public Health, National University of Singapore and National University Health System, Singapore, Singapore

**Keywords:** COVID-19 lockdown, Public health risk communication, Social vulnerability, Public health ethics, Singapore

## Abstract

**Background:**

Mobility restriction is the most effective measure to control the spread of infectious disease at its early stage, especially if a cure and vaccine are not available. When control of the coronavirus disease 2019 (COVID-19) required strong precautionary measures, lockdowns were necessarily implemented in countries around the globe. Public health risk communication about the justification and scope of a lockdown was challenging as it involved a conflict between solidarity and individual liberty and a trade-off between various values across groups with different socioeconomic statuses. In the study, we examined public responses to the government-announced “circuit breaker” (a local term for lockdown) at four-time points in Singapore: (1) entry, (2) extension, (3) exit of lockdown ‘phase 1’ and (4) entry of lockdown ‘phase 2’.

**Methods:**

We randomly collected 100 comments from the relevant articles on new organisations’ Facebook and Instagram pages and conducted preliminary coding. Later, additional random 20 comments were collected to check the data saturation. Content analysis was focused on identifying themes that emerged from the responses across the four-time points.

**Results:**

At the entry, public support for the lockdown was prevalent; yet most responses were abstract with uncertainty. At six weeks of lockdown, initial public responses with uncertainty turned into salient narratives of their lived experiences and hardship with lockdown and unmasking of societal weaknesses caused by COVID-19. At the entry to phase 2, responses were centred on social-economic impact, disparity, and lockdown burnout with the contested notion of continuing solidarity. A temporal pattern was seen in the rationalisation of the lockdown experience from trust, anxiety, attribution of pandemic and lockdown, blaming of non-compliant behaviours, and confusion.

**Conclusions:**

The findings indicated a temporal evolution of public responses from solidarity, attribution of the sustained pandemic, increasing ambiguity towards strong precautionary measures, concerns about economic hardship and mental well-being to worsened social vulnerability, where the government’s restrictive policies were questioned with anxiety and confusion. Public health risk communication in response to COVID-19 should be transparent and address health equity and social justice to enhance individual and collective responsibility in protecting the public from the pandemic.

## Introduction

In the coronavirus disease 2019 (COVID-19) pandemic, various precautionary measures of mobility restriction have been enacted across countries, including travel restriction and ban, quarantine, isolation, restriction of gathering, closure of public places, and lockdowns. Social distancing, which aims to reduce social contact with infected or “at-risk” individuals, was found to be an effective containment measure in infectious disease outbreaks, like influenza pandemics [1]. Thus, it has been one of the primary forms of outbreak control in the early stage of COVID-19, along with screening and treatment while vaccine trials were under development [2]. Non-pharmaceutical interventions – including case isolation, voluntary home quarantine by active case finding and extensive contact tracing, and closure of schools and workplaces – have resulted in the reduction of COVID-19 transmission, mortality rates, and healthcare burden [3–5].

Although strong precautionary measures of mobility restriction are effective, they unavoidably pose potential harm to society broadly. Such measures used in the COVID-19 pandemic could exacerbate social and health inequalities, increase insecurity from economic decline and actual loss of jobs, strain social and family relationships, and eventually expose people to psychological distress and other health-related adversities [6]. These impacts would be greater in the communities with limited resources and protection. Mobility restrictions might be incompatible with meeting essential needs among disadvantaged populations; therefore, they could be more susceptible to COVID-19 [7, 8]. Those confined for a long time by being categorised as “high-risk” groups, like migrants and refugees residing in communal dwellings with high density, are also vulnerable to the stigma attached to the pandemic, which becomes an additional barrier to quality treatment [9]. Due to a broader social-economic impact of the pandemic, policy to contain COVID-19 must be paired with an equivalent social safety net and psychosocial support systems, such as the delivery of essential needs, temporary financial assistance, and parallel planning for rebuilding social capital [10]. It is crucial to note that all these issues and tensions by the control of infectious diseases outbreak, therefore, necessitate well-coordinated and sensible communication on health policies and monitoring of public sentiment reacted by policy changes to ensure proper understanding of these measures and sustainable preventive practice in the public.

The current study explored the temporal schemes of public responses to the lockdown policies in Singapore. The island city-state was reported as one of the most successful countries in controlling COVID-19 through measures of travel ban and lockdown at its early stage [11]. However, migrant workers were disproportionally infected, with a prevalence rate of about 17% compared with 0.04% in the local population [8]. The government implemented a “circuit breaker,” a local term for lockdown on 7 April 2020, for two months. The lockdown was effective. While the COVID-19 vaccination started on 30 December 2020, no more than five daily infection cases have been reported since 7 October 2020 [12]. There were four milestones of policy announcements regarding the lockdown: entry, extension of lockdown with the introduction of a phase-based lockdown exit plan, exit of lockdown, and entry of phase. The study examined how the public reacted to the policy announcements and how their narratives were changed over the four-time periods.

### Public health risk communication in the pandemic control

For precautionary measures to be implemented successfully while minimising potential harms, voluntary public participation is crucial and should be sustained for an extended period, often longer than the public expectation. Public conformity with the government’s measures implemented at the early stage of an outbreak, which was deemed little supported by sound scientific evidence, requires public health risk communications with convincing and reasonable justification. Such communications need to be timely, open, and transparent when managing public health emergencies such as the COVID-19 pandemic [13]. The World Health Organisation (WHO) refers to risk communication as a vital part of outbreak management and advocates for a balance between achieving public health objectives and mitigating disruptions to society, with an emphasis on building and maintaining public trust [14].

In communicating with the public on the risk and consequences of COVID-19 if not controlled, the policy needs to be carefully deliberated with ethical considerations. Decision-making should be transparent, not influenced or coerced by political interference or stakeholders, [15] while the degree of restrictions on individual liberty should be proportional to the degree of expected benefits from public health interventions [16]. The process and efforts of policy development need to be continually and properly communicated to the public so that the public is prepared to buy in and comply with the measures. While transparency is necessary for fostering public trust, being transparent with information to the public could involve disclosing and acknowledging the uncertainty behind unknown scientific evidence and healthcare resource preparedness and the policy decisions that follow [17]. Under such a circumstance of “weakness” in the pandemic control, strong paternalistic measures, like lockdown, could be questioned along with the notions of intrusion of liberty to distributive injustice, which could impede the level of public conformity or acceptance of measures [18].

The news and social media, like social network sites (SNS), play an essential role in not only disseminating health information but also framing it – the way in which a health threat is described and characterised in the media influence the audience’s perceptions of it [19] Infectious disease outbreaks present a scenario where narratives are shaped by discourse directed by governments, constructed in the news, and emerged in the SNS media. The heightened sense of alarm from various stakeholders may allow for the emergence of public health viewpoints that reflect specific political ideologies [20]. Literature described the roles of framing in how the media attempted to form public sentiment and societal values towards pandemics, epidemics, and outbreaks of infectious diseases, like HIV/AIDS, SARS, H1N1, and Zika [21–23]. In the literature, several points should be noted. First, framing was often located in the discourse of emphasising individual responsibility and punishment, if not compiled, rather than structural and social determinants of health vulnerability [24]. Second, temporal changes in the coverage of pandemics are largely outcome-based, either case reports or policy changes, to yield ‘better outcomes’ rather than procedural and distributive justice [25]. Third, framing was not merely unidimensional and directive but involved multiple sectors at stake, including the public, who re/de-constructed frames in response to top-down communication [26].

In the COVID-19 pandemic, increasing studies reported public sentiment and responses to precautionary measures in countries, including China, [27] India, [28] the Philippines, [29] Italy, [30] and the US [31]. These studies focused on the responses primarily drawn from popular SNS like Twitter and Facebook but did not examine temporal patterns of the public responses, despite the development of the pandemic and the policies toward it changing over time. Thus, current empirical studies on health communication and public responses to COVID-19 are mostly limited to descriptive analysis at a given time point. To better understand the construction of public sentiment and health communication in the changing pandemic, there is a need for research on the temporal changes of public responses to COVID-19 policy from the perspective of risk communication and ethical considerations on varying impacts of restriction measures.

**Fig. 1:**
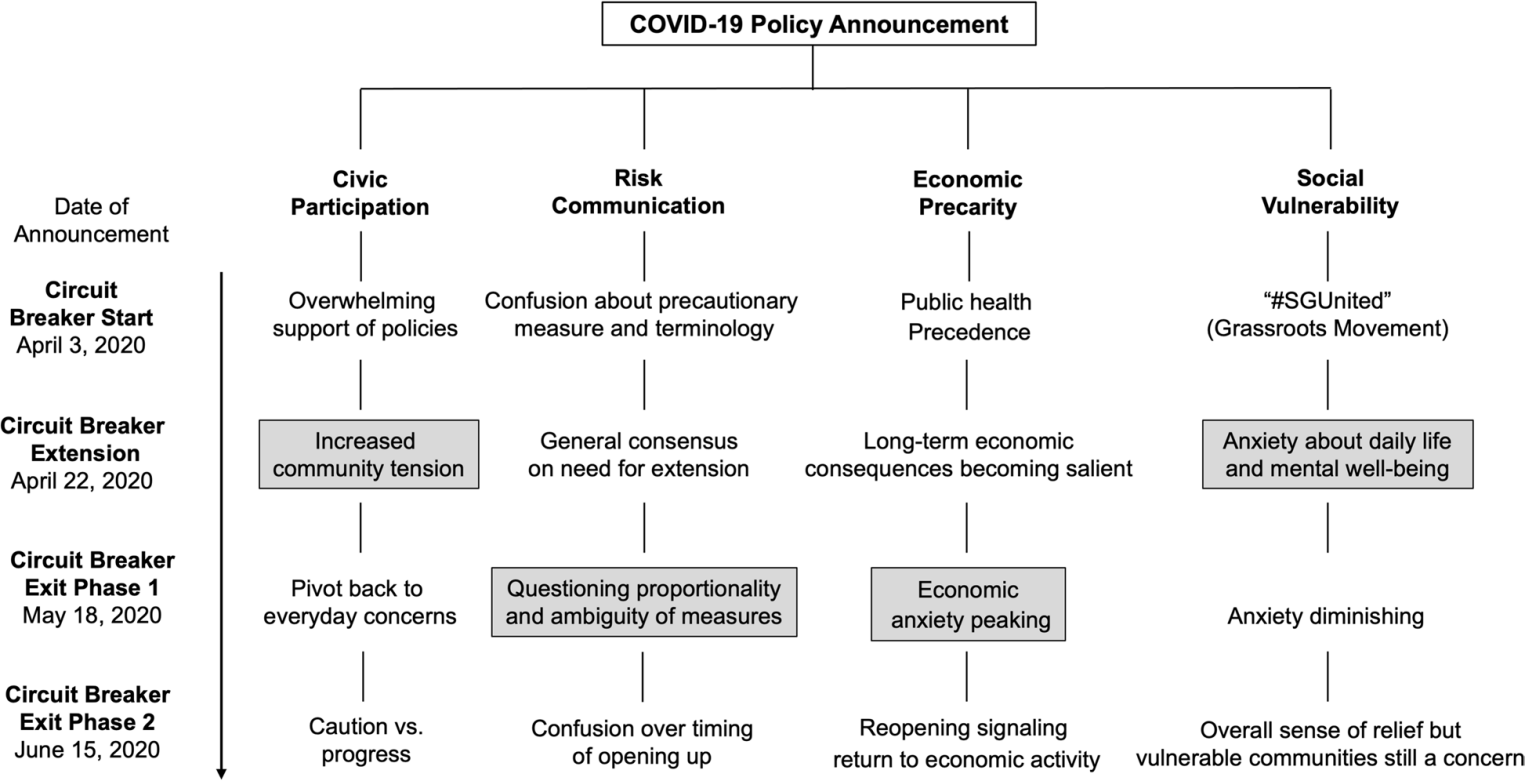
Temporal Evolution of the Main Themes Through the Different Lockdown Periods

Inquiry of policy discourse through SNS is especially important to ascertain public sentiment and stance in Singapore, where a strict and unwavering restriction of public demonstrations was regulated [32]. Less-regulated SNS is widely used to voice public opinions while promoting civic activities [33]. Our study aim is twofold. First, we qualitatively examine what public responses were when the government announced the “circuit breaker” at the four-time points: (1) entry, (2) extension, (3) exit of lockdown (‘phase 1’) with the announcement of a three-phase lockdown exit plan and (4) and entry of lockdown ‘phase 2’ (see Fig. 1 below). Second, we explored how the public responses evolved over four phases and the temporal patterns in emerging themes. Public SNS responses to news reports covering these COVID-19 policy changes represent an immediate set of reactions to the containment measures and demonstrate citizen perspectives in the absence of physical avenues for civic engagement. We used the notion of framing as a communicative scaffold that is collectively built by organic SNS reactions to policies. Lastly, there is no current literature on the temporal media analysis of public opinions and sentiment toward the government policies of mobility restrictions that are constantly changing in response to COVID-19 situations. The findings from our temporal analysis would provide insights for future pandemic preparedness policies, especially in terms of risk communication in infectious disease outbreak control.

## Methods

### Data collection

We identified three notable English news organisations in Singapore - Channel NewsAsia (CNA), The Straits Times (ST), and Mothership. As reported in Reuters Institute’s 2019 Digital News Report, CNA and ST are Singapore’s most highly used online news media sources [34]. While still a relatively new organisation, Mothership ranked fourth in online news usage. More importantly, Mothership was chosen to account for responses from younger Singaporeans. As of October 2019, Mothership had a monthly readership exceeding 4.5 million unique users, with two-thirds of readers between 25 and 44 years old. The data collected from this site were to better account for the age demographic. From these news organisations, we selected articles that covered significant government announcements concerning Singapore’s ‘circuit breaker,’ namely: (1) announcement of circuit breaker (April 3, 2020); the lockdown included school and workplace closures and confinement of all migrant workers in dormitories, (2) extension of circuit breaker (April 21, 2020); due to sustained COVID-19 cases, particularly among migrant workers, it was extended to 1 June with stricter measures of closing more businesses, (3) the exit from the circuit breaker to phase 1 (May 18, 2020); the government announced a three-phase lockdown exit plan, with a gradual return to work, school, and community activities: “safe reopening, transition, and nation”, and (4) entry of phase 2 (June 15, 2020) [35]. Table 1 shows the timeline and summary of policy announcements.

**Table 1:** Timeline and summary of policy announcement

Milestone	Summary of Announcement
Circuit Breaker Start 3 April 2020	Singapore Prime Minister Lee Hsien Loong announced the start of Singapore’s ‘Circuit Breaker’. Policies announced: • One-month duration • Most workplaces closed • Full home-based learning in schools • Tighter movement restrictions
Circuit Breaker Extension 21 April 2020	Singapore Prime Minster announced the initial two-week extension of the Circuit Breaker to “decisively” bring down the number of cases. Policies announced: • Closure of more workplaces/businesses • Stricter entry restrictions • Only one person should be away from home at any one time • Mandatory mask-wearing
Circuit Breaker Enter Phase 1 18 May 2020	The exit from the Circuit Breaker into Phase 1 was announced to the public. Policies announced: • Some businesses will reopen, but most will stay closed • Places of worship can reopen for private worship only • Households can only have two visitors a day who must either be children or grandchildren from the same household
Circuit Breaker Enter Phase 2 15 June 2020	The transition from Phase 1 to Phase 2 was announced to the public. Policies announced: • Group sizes increased to five • Households can have five visitors • Most public facilities can reopen • More businesses can reopen • Dining-in can resume

Public responses in English were collected from the relevant articles uploaded on each news organisations’ Facebook and Instagram pages, except for CNA, which used its Instagram page exclusively for lifestyle news. Additional responses were randomly collected from posts by the Prime Minister’s Office. These responses are publicly available in the SNS comments section. Responses were selected if they directly addressed the announcements, for example, “We should have started wearing masks earlier.” In addition, comments that only included emojis were excluded from the sample as well. We initially randomly collected 100 comments from each article and conducted preliminary coding. Later, we added another random 20 comments to compare the existing codes to check theoretical saturation – whether there was a new theme identified from the additional data. During the coding of the additional 20 comments to check the data saturation, we found a few comments that might not fit among the major four themes, such as “questioning the accountability of WHO” and “comparing other countries’ lockdown measures.” However, as there was no temporal evolution in the themes, we did not create another theme relating to the comment; instead, we put them under the theme of “risk communication”. Other than this, no additional theme was identified, and saturation was confirmed.

### Qualitative thematic analysis

The comments collected were entered in NVivo 12 for data management and centralising data analysis. We used thematic analysis based on a grounded theory approach for coding and creating themes [36]. Firstly, all three authors read the data to immerse themselves in its context, noted down initial ideas of open coding, and then shared notes with each other. Next, the first and third authors conducted open coding of the data from the first media sources independently, creating broad initial themes that were later consolidated with the second author. After constructing the coding structure, we completed coding of the rest of the data from two other sources following the same process. This process of two independent coding, which was checked by the second author, ensured the credibility of analysis. After the finalisation of open coding, the three authors performed focused coding to collate codes into more specific themes and generated a ‘thematic scheme’ of the analysis where themes were named and refined to fit into the overall narratives of the study research question.

## Results

We identified four main themes, each with four sub-themes that correspond to specific announcements during the lockdown. Table 2 presents the thematic schemes. While the main themes – civic participation, risk communication, economic precarity, and social vulnerability – are presented under each category, it is important to note that they are not independent as their interrelatedness and intersectionality constructed public sentiment as a whole.

**Table 2:** Hierarchal Thematic Scheme of Public Responses to COVID-19 Policy Announcement

Themes	Sub-themes
Civic Participation	Community participation was framed as a form of digital civic participation: • Overwhelming support • Increased community distrust • Pivot to everyday concerns • Caution vs progress
Risk Communication	Inconsistency in disseminated health information caused debates in comments: • Confusion over public health instruction • Consensus on necessity of the circuit breaker • Proportionality of public health decisions • Scepticism over policy frames and decisions
Economic Precarity	As the lockdown necessitated a pause on economic activity, comments progressively addressed: • Economic vs public health precedence • Salient economic consequences • Peaking economic anxieties • Return to normalcy
Social Vulnerability	As the lockdown progressed, comments progressively addressed societal weaknesses: • #SGUnited (grassroots movement to help vulnerable populations) • Anxiety about low-income livelihoods, mental health, and older adults • Non-inclusive protection of migrant workers

Figure 1 illustrates the overall temporal evolution of the main themes through the different lockdown periods, with every subsequent sub-theme indicating the dominant discursive topics in the comments. Themes highlighted in rectangles represent the most salient sub-theme throughout the progression.

### Civic participation

Through online engagement, citizens actively participated in the government’s lockdown policy, expressing their direct support for precautionary measures of mobility restriction, indirect suggestions, questions on effectiveness, and disapproval. Discussions included consensus-driven, attribution, and rationalisation behaviours in response to policy announcements. Such civic participation reached its peak after the announcement of the extension of the lockdown, where minority voices questioning the policy increased and created tension against pre-existing dominant voices of full support.

#### Lockdown start

Commenters expressed overwhelming support for the lockdown. This support manifested in several ways. Many commenters directly thanked the government and expressed support for the policy. If commenters did not explicitly express their gratitude, they showed support through policy suggestions. Specifically, many even called for stricter measures on top of the ones already introduced by the government to control the mobility of community members. Measures suggested including a “temporary tobacco sales ban,” “stop people from buying (groceries) from the supermarket directly,” and even heavier fines or jail sentences for those who did not comply with social distancing measures. Many comments on civic surveillance were posted.

*I saw several women exercising together every morning in the playground. I suspect that they mingled around after the workout. Should I report this to the authority to disperse them?*

As such, there was a noticeable attribution of blame toward non-compliant community members for the worsening situation. Some commenters even used these comment threads as indirect whistleblowing channels to highlight non-compliance within the community, ranging from inadequate mask-wearing to food vendors’ lacking adequate precautionary measures. One person went to the extent to claim that they would participate in community enforcement, saying that he was willing to be a “soldier of the community.”

*Thank Prime Minister! My family and I assure you that we will be at home during this tough period and pray for Singapore and its community. I am also ready as a soldier of this community [emphasized by the commenter] if anything is to be done by myself if required by the government.*

Many commenters, most of whom were older adults, personally and directly addressed other community members online, acting as informal public health messengers for the government. They urged for greater social responsibility and reiterated compliance with government measures for the sake of the country.

#### Lockdown extension

The lockdown extension had prompted increasing complaints on individual behaviours, which were regarded as “unacceptable” according to the lockdown measures.

*You all leave your house for no valid reasons, then when the circuit breaker is extended, you all want to get mad. You all stay at home and obey the rules. It is not fair to those who stayed at home while others go out to buy food or everyday needs. Damn unfair. Think about all of us.*

Comments of individual blaming and naming increased, which led to community tension and eroding community trust. Many commenters were especially frustrated with “(un)cooperative” and “stubborn” community members, citing multiple anecdotes of non-compliance. They also attributed the extension of lockdown to those individuals. While sparsely used, the emergence of the term “#COVIDIOTS” during this period aptly encapsulated the increasingly tense state of community solidarity. In response, calls for stricter “iron-fist” policies to control mobility in the country intensified, marking an increase from when the lockdown started. These sentiments and online behaviours are characteristic of online rejections of deviant behaviour or forms of “digitalized vigilantism,” a process of online coordinated retaliation against offensive citizen activity [37]. This was not unexpected, as collectivist societies like Singapore, with their emphasis on social responsibility and social consequence, are more likely to punish those who threaten the collective welfare of the community.

#### Lockdown phase 1 start

The announcement of phase 1 marked a shift of commenters’ discussion toward other main themes, like the emergent economic impact of loss.

*Why so many phases? Why can’t we just totally shut down for two weeks and reopen? We have already gone so far with all the effort and sacrifices.*

Narratives of solidarity and individual responsibility from the previous phases decreased significantly, with attention turned toward themes related to the resumption of everyday life. However, discussion about the country’s readiness to transit out of the lockdown began to rise, with many indicating anxiety about opening too soon. Some commenters expressed the same distrust in fellow community members seen in the previous announcements, worried that irresponsible community members would cause another spike in cases. While some commenters then asked for continued social responsibility, others wanted a second extension. This marked another point of tension.

#### Lockdown phase 2 start

The public anxiety and tension between precaution and wish for reintegration increased on the announcement of phase 2.

*The current situation has gotten bad enough already! I cannot imagine how phase 2 is going to work out. I am speechless.*

While some felt that it was too soon for mobility restrictions to be lifted, their wish to go back to normal had them welcome ideas of reopening.

*There's a thing called choice. I believe it's a good time to open up, at least as a test since it has been closed for so long. We opened then we can see the results. You never know until tested.*

After experiencing the lockdown and observing the decrease of COVID-19 cases, there was a tendency for commenters to shift the responsibility from the government to individuals – the government had made vast efforts on public health precautionary measures; therefore, individuals must have been educated and aware of the importance of compliance. However, several comments with scepticism were seen in terms of the ability of community members to continue to follow restrictions upon the lifting of the current restrictions.

*How are they going to enforce safety regulations when people are already taking things so lightly in phase one? [crying emoticon]*

The need for collective responsibility was once again emphasised as many commenters doubted the efficacy of social distancing once the harshest measures were lifted.

### Health risk communication

Citizens’ stance on the government’s lockdown policy was closely related to how they were involved in the risk communication of COVID-19. Health communication in a pandemic aims to earn public trust and develop a base for increasing social cohesion and solidarity against public health threats. In the early stage, public health communication did not adequately address the uncertainty of health risks relating to the outbreak of COVID-19. There were questions about the issue of mask-wearing and mistrust in certain policies, most apparent during the transitory periods, namely the start of lockdown phase 1. Rather, they expressed various levels of uncertainty regarding the accuracy, transparency, and purpose of the information presented.

#### Lockdown start and extension

While there was pervasive support for the lockdown, many commenters expressed frustration at initial policy inconsistency and informational ambiguity. This raised doubt about the overall reliability and adequacy of the risk information provided.

*Why need to wait until the number keeps increasing then take action? Can’t see that lockdown is already too late? Why need to wait until the evidence is out? News from other countries already reports that persons with no symptom of the virus still pass it on to others. Our country is the same as family. We need to take care of ourselves first instead of looking for your own benefits to keep inviting problems. Now problems are arising. Do we need to enforce all of these because of your mistakes?*

Commenters raised concerns about the lack of information on asymptomatic cases in the community. This ties into many commenters’ sentiment about inconsistent government measures, with many commenters referring to the actions as “prata-flipping,” a local colloquial describing indecisiveness. Commenters specifically pointed out the government’s relatively late response on mask-wearing, which many regarded as an intuitive public health policy of which “scientific evidence was… there all along”. While some noted that such policies were made based on available evidence, commenters were still largely critical. Relatedly, commenters expressed ambiguity regarding the sources of policymaking, such as other countries’ cases and WHO indicating increased scepticism towards the global health institution. Some commenters were not satisfied with the government’s soft approach of merely “urging” the public to follow the rules, preferring that these recommendations be made compulsory.

#### Lockdown phase 1 start

Confusion among commenters was at its most intense on the announcement of phase 1, which was introduced with many restrictions on social distancing and gathering in public and private places.

*Private worship means what?? Can we have worship in a residential place (private worship)? See not clear again!”*

The confusion over the specifics of reopening measures was predominant. There were debates on the definitions of restricted places and actions due to the perception of lacking explanation for policy decisions. During the lockdown phase, social distancing orders were perceived as relatively straightforward. In particular, commenters questioned why mobility restrictions were still strict despite cases being low. They reemphasised the government’s apparent policy contradictions, with some attributing it to the government being too “kiasu” or overly cautious.

*For 95% of people in our country, Phase 1 is just another four weeks of lockdown extension.*

For most of them, a stepwise lift of lockdown was difficult to comply and connect with. While the announcement was made as a transition away from lockdown, the unchanged movement restrictions gave them an impression of little difference. The ambiguity in risk communication messages was still salient.

#### Lockdown phase 2 start

There continued to be uncertainty about the policy of entering phase 2.

*Phase 2 opening amid ongoing new imported cases? Oh well...whatever fits your convenience. COVID-19 never ends without other countries' end.*

The timing of phase 2 was commented as arbitrary and ambiguous. There was no clear evidence of how the lockdown policy was supported by evidence – to what degree the pandemic situation determines the change of social distancing policy. Some commented that the policymaking was rather political, not scientific, that phase 2 was put into place to allow the government to hold an election in a time of crisis.

### Economic Precarity

With the public forced to stay home, the lockdown undoubtedly disrupted and affected economic sectors. At the first point of the lockdown announcement, the economic impact was expected and viewed as a necessary sacrifice; therefore, there were few discussions on economic anxieties. However, the fear of financial loss increased from the announcement of the extension and peaked from the announcement of the exit plan.

#### Lockdown start

In line with the wide support for the lockdown, public health goals took precedence over the potential economic consequences from restrictions. Commenters overall agreed that saving lives was more important than financial loss, “economic can get back, but life cannot be.”

*If the government wants a full lockdown, let’s do it. Don’t mind the economic failures and so on. Those lives working on the essential sectors are also important. I believe Singapore can build the economy back again. Don’t wait for it to get really too bad.*

Since the lockdown was framed as a last resort to curb the spread of COVID-19 transmission, commenters were generally willing to take the risk of economic consequences brought on by the lockdown. The government’s compensation of $600 to every citizen announced three days prior to the lockdown appeared to help public conformity [38].

### Lockdown extension

After the announcement of the extension, there was a noticeable shift of comments from public health cooperation to salient anxiety about the future economy.

*Please tell us how the government is going to assist us further? There are bills and instalments to pay. Extension till June means another month of no income for some.*

While commenters still largely agreed that the extension was a necessary sacrifice and phase-approach was reasonable, increasing worry over its economic effects became more apparent, though not reaching a peak until the announcement of phase 1.

#### Lockdown phase 1 start

While Phase 1 was described as a transition out of the lockdown, commenters disagreed and were sceptical about the actual effects of the transition as there were still strict measures of work-from-home and restrictions in commercial sectors.

*No matter whatsoever, this circuit breaker has to end. We have to resume our economic sectors soonest possible. A lot of those working adults are facing pay cuts and retrenchment.*

While the macroeconomic impact of the pandemic was well known, a substantial increase of comments on the individual loss of jobs and profits was pervasive. Following the extension of the lockdown, commenters felt that businesses would not survive for much longer, and their main concerns were when the lockdown would be completely lifted, separate from the question of when the pandemic would end.

*The government should have economic empathy and allow all businesses, including retail and entertainment outlets, to resume from Phase 2 onwards as long as they can adopt safety measures. If the government needs two to three months before resume, the government needs to tell them to close shops now, so they retrench all their workers and cut losses.*

Many felt that the “slow” opening would further debilitate local businesses and jobs. In particular, this economic anxiety was highly escalated among lower-income households, who are mostly involved in essential services.

#### Lockdown phase 2 start

Expectedly, once phase 2 was announced, these anxieties were alleviated as there was hope that businesses could resume and revive.

*We cannot be under lockdown forever. We need to handle this the right way. Practice good hygiene and wear your masks. We have to go back to our everyday lives someday; that has to be soon. The economy has to pick up as well... Thank God*

Such relief was shared among commenters. Their wish to return to normality was narrated with mutual support for commitment to practising precautionary measures, such as QR entry check-in, masking wearing, hand washing, and limited social gatherings. The lockdown brought about economic loss, but public health education on COVID-19 prevention seemed to have percolated amongst the public.

### Social vulnerability

As the pandemic affected communities at risk of health-related adversities and the lockdown continued, comments on social vulnerability emerged and intensified on SNS, where commenters highlighted societal weaknesses amongst the country’s vulnerable populations. With jobs lost and social ties severed, the pandemic unmasked and exacerbated problems that were previously ignored, unsaid, or unseen by the community. The salience of social vulnerability appeared to depend on the level of isolation expected from each lockdown progression. As social isolation became an increasing reality, commenters were more concerned about it and specified the situations.

#### Lockdown start

Due to the public health urgency, commenters rarely raised any societal issues in the community. Instead, they appealed to solidarity and social responsibility for the pandemic.

*It is not the responsibility of the government alone. It is that of the entire nation, making it the duty of every Singaporean to do their part. “SGUnited,” we will prevail.*

Besides the nascent mention of potential financial difficulties for lower-income families and older adults, most of the comments were focused on public health risks and conformity.

#### Lockdown extension

On the announcement of the lockdown extension, these unsaid concerns increasingly appeared in the comments and became the most salient.

*If our help does not reach people, especially those who are jobless, or their job search is bleak despite being proactive, we must be prepared to deploy more resources on the ground level.*

Firstly, comments on the mental health consequences of extended time at home intensified.

*For a person with depression extending the circuit breaker is not going to help. But only will it make the depressed person worse if the problem is started at home. Now being stuck with the people who developed depression is extremely hard.*

Commenters cited issues such as depression in the vulnerable communities of older adults, lower-income families, and essential workers. For older adults, this was mainly attributed to the lack of social support. For people with lower income, it was attributed to the loss of jobs and lack of a stable income which became more urgent due to utility bills.

*Please extend more help to older adults living alone, those living in rented flats, and families with low income! They are the most affected by COVID-19.*

A few commenters felt that the government’s decision of a lockdown extension did not sufficiently address the mental well-being of the citizens despite the necessity of the extension itself being widely agreed upon. They also suggested how social protection should be included in evaluating the efficacy of public health policies. COVID-19 cases were mostly concentrated among low-wage migrant workers who resided in unhygienic and high-dense dormitories, which sparked debate on how much care should be given to the population.

*Why do we local Singaporean have to bear the consequences of mobility restrictions, whereas the actual COVID-19 cases now are all coming from migrant workers’ dormitories?*

Some commenters expressed sympathy for migrant workers, and others wondered why the local community had to suffer from COVID-19 cases “outside”. In this case, public health communications were used to frame the discourse of creating the boundary with “others.”

#### Lockdown phase 1 end and phase 2 start

Comments on these societal weaknesses decreased from the announcement of the phase 1 exit. The exit policy of allowing, but holding, familial visits to five persons helped address social isolation and loneliness among older adults. While commenters paid special attention to social vulnerability, which requires structural intervention, they also appealed to a sense of collectiveness.

*If we can practice social distancing, wear a mask and wash hands (simple, right?), we can do it. Please, #covidoits, sovereigns don’t make trouble. It took us so long to see some sense of normal.*

This calling for collective responsibility summarized their wish for a return to normal.

## Discussion

This study highlighted the changing narrative patterns of SNS comments in response to Singapore’s COVID-19 policies of lockdown and following mobility restrictions. Overall, our temporal analysis indicated transitions from solidarity, attribution of the sustained pandemic, increasing ambiguity towards precautionary measures, economic hardship, mental well-being to social vulnerability, where government lockdown policies were questioned and met with anxiety and confusion. While immediate public reactions to the lockdown implementation were agreeable, the comments featured abstract indications of an uncertain future, which were suppressed and masked by the initial urgency of the pandemic. These uncertainties eventually manifested themselves in worries about the economy, social and individual security. There had always been a tension between pandemic control and social concerns. While commenters’ reactions towards the lockdown extension were largely marked with increased community tension, increased anxiety about emergent weaknesses, and growing salience about economic consequences, there was still the consensus on the need for mobility restriction and its extension. When the lockdown exit plan was announced, the potential return to normalcy eased heightened anxiety. However, this coincided with more complaints about the proportionality and ambiguity of the measures in the subsequent phase. The findings emphasised that the public health risk communication strategy that benefited from authoritative information at the beginning of the epidemic needs to deliver more evidence-based policy measures and transparency in justification for decisions.

At the start of lockdown, Singaporeans were largely supportive of the policies, largely underpinned by a ‘public health emergency.’ Despite many of the policies exhibiting elements that could be considered authoritarian or draconic by citizens, these were viewed as necessary sacrifices for the nation’s sake. Such conformity by Singaporeans was reported from previous outbreaks. A survey conducted during the SARS outbreak found that the majority of Singaporeans were willing to change their behaviours, even if it meant drastic modifications to their life [39]. The survey also found that financial impact on the Singapore economy ranked after respondents’ concerns for individual health and society. Even when discussing the uncertain future of the economy and others’ livelihoods in the subsequent phases, these worries were accepted as an inevitable consequence. There were hardly any comments that called for the modification of mobility restriction elements to accommodate the needs of the communities most affected. Rather, the safety of the country was the top priority.

Another notable finding is how collective calling for solidarity can be linked with (public) blaming certain individuals. Despite the initial hopeful, collectivist, and enthusiastic overtones of “social responsibility” and public trust at the start, citizens’ proclivity to blame fellow citizens became apparent as the lockdown progressed. Vindictive attitudes in public were intensified with sustained social distancing. A formation of digital vigilantism emerged in the comments in response to defiance against the lockdown rules. This phenomenon was illustrated by a Facebook group, tagged as “SG COVIDIOTS,” and its many other variations, where the public uploaded pictures or videos of non-compliant citizens as a form of civic engaging enforcement. As Trottier (2017) argued, this vigilantism potentially acts as a criticism of the government policy of restriction measures [37]. Thus, such acts could covertly weaken solidarity during the lockdown period, which presents additional deleterious social consequences of isolation policies on top of the effects [6].

The finding from the analysis of risk communication suggested that the communicative and informational apparatuses of the government did not adequately address public health uncertainty evoked during the course of the pandemic. The key challenge of communicating public health uncertainty lies in how it conveys ambiguity in the information provided [15, 40]. At the early stage, the public was uncertain about preventive measures and, later, certain rules of restrictions concerning business and social gatherings. These uncertainties created doubt in the public about the overall integrity and adequacy of the risk information provided. Ambiguity in public health policy could provoke psychological responses such as “ambiguity aversion” when the public avoids choices that present certain levels of uncertainty [40]. Transparent government communication of the uncertainty in health risk, mainly due to a lack of scientific evidence, could alleviate public anxiety and ambiguity.

Public awareness of societal weakness and the impact of the prolonged restriction on vulnerable populations was notable. In addition to the goal of reducing COVID-19 infection and its related morbidity and mortality, public health policy should be established with another goal of protecting affected vulnerable populations. Specifically, the goal is to reduce psychological distress, social isolation, and stigma caused by the pandemic. Disadvantaged populations are more exposed to social stressors without proper coping resources; therefore, they experience more mental illness. Public mental health considerations should be incorporated in infectious disease control policy. Structural causes of social vulnerability will remain or be further deepened if the harms which are caused by COVID-19 are not reduced. Long-term consequences of socioeconomic disruptions need to be addressed post-COVID-19.

There are limitations to the study. The study mainly used comments from Instagram and Facebook. All the comments collected were in English, failing to capture segments of the population that may not be fluent in English. Users of these sites may not be representative of the overall population. Other data collection methods, such as surveys, phone inquiry or community engagement, would be necessary to increase representativeness. Comments were drawn from comments on SNS posts by news outlets announcing specific periods of lockdown measures. They did not include user-created content and news reports on other specific lockdown-related matters, such as reports of violations or new advice on social distancing, mask-wearing, school closures, and other containment measures. We did not find novel views from user-created content like blogs. All their views were narrated from our datasets of 360 comments. The dataset, therefore, captured the immediate responses of citizens to the various measures rather than the long-term sentiment regarding health policy and communications decisions.

## Conclusions

The COVID-19 pandemic has highlighted the importance of public health risk communication, which should be transparently, clearly, and accurately conveyed to the public. While the state’s risk communication in response to the pandemic emphasised collective responsibility, the public raised questions on lacking specificities in policy communication. Increasing ambiguity throughout the lockdown period could facilitate burnout with prevention measures, especially when essential social and economic needs were unmet among vulnerable populations. The findings of such contested solidarity and vulnerability underlined the complexity of COVID-19 public health threat. The solution to it involves a trade-off between many values at stake across different groups in society. While the “best” practices of pandemic policy could not be empirically tested due to different values and outcomes in local contexts, social justice as health equity and inclusive protection beyond mere disease control needs to be put into the context of public health risk communication. This will eventually enhance individual and collective responsibility in protecting the public from the pandemic.

## Data Availability

The datasets used and/or analysed during the current study are available from the corresponding author on reasonable request.

## Abbreviations

COVID-19: Coronavirus disease 2019
HIV/AIDS: Human Immunodeficiency Viruses/Acquired Immunodeficiency Syndrome
SNS: Social Network Sites
SARS: Severe Acute Respiratory Syndrome
H1N1: H1N1pdm09 virus
